# Cast Boxes for Soldering in

**Published:** 1848-07

**Authors:** 


### CAST BOXES FOR SOLDERING IN.

Our friend Leslie brought on from New York, where he had picked up the idea from Dr. Parmly a pattern of a semi-circular casting, about an eighth of an inch thick, with a rim about one inch in height, and a bottom about one inch wide. Several sizes are made, varying from three to four inches across the point, the bottoms also varying in width and narrowing as it runs back to the point. Several sizes are convenient for the different sized jobs; Perhaps a more familiar notion may be obtained by comparing its shape to the caps used generally in taking up impressions without their handles or inside rim. When the job, a full or partial row is ready for the plaster which is to be mixed up pretty thick, one third to one half clean sand, a portion is poured into the bottom, thick enough to keep the plate or teeth from touching the iron; the job is then let in the labial surface to the rim, leaving from an eighth to a quarter of an inch to fill up with the paste, the edges of the teeth may be covered up and the teeth lined without being taken out. The borax and solder is then applied, when the whole is put into the fire and heated until the iron becomes red, it is then taken out and applied to the blow pipe, when very little more heat will be requisite to solder the whole job. A very convenient perquisite to this is to have another similarly shaped, which may be made of sheet iron, the edges notched out so as to turn up for the rim, and the points being again turned over the edge of the rim, will prevent it from falling out; leave the centre of the bottom a little prominent to put a pivit through, and through the end of an iron handle, not riviting it so tight but it will turn around; in this manner it may be so turned about as to direct the heat to it in any position. We think that any dentist who has once used them will never be without them. If the plaster be wet it must be heated up gradually, or the rapid evaporation will throw it out of the box. With new boxes care must be observed and not let the job remain in too long, or the oxide of the iron will strike through and discolor the teeth.

Being made of cast iron they cost but little. Dr. Brown, of our city, who has almost every thing the dentist can think of or desire, and deals them out in most accommodating manner and terms, intends adding a good supply of these to his stock. E. T.

